# Analysis of Electronic Health Record Use and Clinical Productivity and Their Association With Physician Turnover

**DOI:** 10.1001/jamanetworkopen.2021.28790

**Published:** 2021-10-12

**Authors:** Edward R. Melnick, Allan Fong, Bidisha Nath, Brian Williams, Raj M. Ratwani, Richard Goldstein, Ryan T. O’Connell, Christine A. Sinsky, Daniel Marchalik, Mihriye Mete

**Affiliations:** 1 Department of Emergency Medicine, Yale School of Medicine, New Haven, Connecticut; 2 Department of Biostatistics, Yale School of Public Health, New Haven, Connecticut; 3 MedStar Health National Center for Human Factors in Healthcare, Columbia, Maryland; 4 Northeast Medical Group, Yale–New Haven Health, Stratford, Connecticut; 5 American Medical Association, Chicago, Illinois; 6 Department of Urology, MedStar Health, Washington, DC; 7 MedStar Health Research Institute, Hyattsville, Maryland; 8 Department of Psychiatry, Georgetown University Medical Center, Washington, DC

## Abstract

**Importance:**

Physician turnover takes a heavy toll on patients, physicians, and health care organizations. Survey research has established associations of electronic health record (EHR) use with professional burnout and reduction in professional effort, but these findings are subject to response fatigue and bias.

**Objective:**

To evaluate the association of physician productivity and EHR use patterns, as determined by vendor-derived EHR use data platforms, with physician turnover.

**Design, Setting, and Participants:**

This retrospective cohort study was conducted among nonteaching ambulatory physicians at a large ambulatory practice network based in New England. Data were collected from March 2018 to February 2020.

**Main Outcomes and Measures:**

Physician departure from the practice network; 4 time-based core measures of EHR use, normalized to 8 hours of scheduled clinical time; teamwork, percentage of a physician’s orders that are placed by other members of the care team; and productivity measures of patient volume, intensity, and demand.

**Results:**

Among 335 physicians assessed for eligibility, 314 unique physicians (89.2%) were included in the analysis (123 [39%] women; 100 [32%] aged 45-54 years), with 5663 physician-months of data. The turnover rate was 5.1%/year (32 of 314 physicians). Physicians completed a mean 2.6 appointments/hour (95% CI, 2.5-2.6 appointments/hour) and 206 appointments/month (95% CI, 197-215 appointments/month) with 5.5 hours (95% CI, 5.3-5.8 hours) of EHR time for every 8 hours of scheduled patient time. After controlling for gender, medical specialty, and time, the following variables were associated with turnover: inbox time (odds ratio [OR], 0.70; 95% CI, 0.61-0.82; *P* < .001), teamwork (OR, 0.68; 95% CI, 0.52-0.87; *P* = .003), demand (ie, proportion of available appointments filled: OR, 0.49; 95% CI, 0.35-0.70; *P* < .001), and age 45 to 54 years vs 25 to 34 years (OR, 0.19; 95% CI, 0.04-0.93; *P* = .04).

**Conclusions and Relevance:**

In this study, physician productivity and EHR use metrics were associated with physician departure. Prospectively tracking these metrics could identify physicians at high risk of departure who would benefit from early, team-based, targeted interventions. The counterintuitive finding that less time spent on the EHR (in particular inbox management) was associated with physician departure warrants further investigation.

## Key Points

**Question:**

Can vendor-derived electronic health record (EHR) use data platforms be used to understand and explain physician turnover?

**Findings:**

In this cohort study of 314 physicians in a large ambulatory care network, physician productivity and EHR use metrics were associated with physician departure. Counterintuitively, less time spent on the EHR (in particular inbox management) was associated with physician departure.

**Meaning:**

These findings suggest that prospectively tracking these metrics along with standardizing metrics across vendor products could identify physicians at high risk of departure who would benefit from early, team-based, targeted interventions.

## Teaser

This cohort study evaluates the association of physician productivity and electronic health record (EHR) use patterns with physician turnover.

## Introduction

Physician turnover disrupts patients’ continuity of care, the lives of physicians and their families, and strains health care organizations’ culture, climate, and finances. Physician replacement costs to a health care organization (including recruitment, lost revenue, onboarding, and time to return to optimal efficiency) have been quantified at as much as $1 million per physician departure.^1^ This translates to approximately $4.6 billion annually at the national level.^2^ These costs are particularly worrisome given high rates of physician burnout^3^ and a projected shortfall of 35 000 to 90 000 physicians by 2025.^4,5^

Cross-sectional analyses have established associations between professional satisfaction, burnout, intention to leave,^6,7,8,9,10^ and actual reduction in professional effort.^11^ Given excessive physician time spent on electronic health record (EHR) activities^12,13,14,15^ and its association with professional burnout,^16,17,18^ it is not surprising that physician dissatisfaction with the EHR has been associated with intention to reduce clinical work in the next 12 months (in national sample of 6880 physicians: OR, 1.44; 95% CI, 1.16-1.80; *P* = .001) and to leave one’s current position in the next 24 months (OR, 1.57; 95% CI, 1.27-1.93; *P* < .001).^8^ In particular, EHR inbox message volume has been strongly associated with physician burnout.^19,20^

Survey research is subject to the limitations of self-report, including response fatigue and bias. EHR audit log data has the potential to overcome these limitations by providing an automated, objective data source for measuring physician productivity and EHR use.^21,22^ Recognizing that physician departure is multifactorial (ie, attributed to reasons that are personal and financial and related to work culture, work climate, workload, and so on), we assume an association exists between physician workload and departure. Given this assumption, reliable measurement of physician productivity and EHR use could help to identify physicians at increased risk of departure.^23^ In 2020, 7 core EHR use metrics were proposed to standardize ambulatory physician EHR use measurement normalized to 8 hours of scheduled patient time.^24^ Five of these metrics could be implemented for ambulatory, nonteaching physicians in 2 health care systems operating different EHR vendor products.^22^

To our knowledge, no previous studies have examined physician productivity and EHR use patterns and their association with physician turnover. Therefore, we conducted a study of ambulatory physicians’ turnover across an ambulatory care delivery network over a 2-year period to determine the association of physicians’ productivity and EHR use patterns with departure. Based on their positive association with physician burnout,^19,20,25^ we hypothesized that increased time on EHR activities (in particular inbox management) would be associated with higher rates of physician departure.

## Methods

### Study Design and Setting

This retrospective cohort study of ambulatory physician turnover examined physician turnover, productivity, and EHR use from March 2018 to February 2020 in a large ambulatory practice network in New England. Given known challenges implementing core EHR use metrics with vendor-derived data in the previous implementation of the 7 core metrics for ambulatory physician EHR use,^22^ the study cohort was limited to the ambulatory division of a practice network that employs ambulatory-based physicians who are out of training and have no teaching responsibilities. The network includes 141 practice sites in Connecticut, New York, and Rhode Island and operates on a single installation of the Epic EHR (Epic Systems). The study protocol was approved by Yale University’s institutional review board. Informed consent was waived, as all study data were deidentified, no private health information was collected, and obtaining consent would jeopardize the scientific validity of the findings given the likelihood of introducing participation bias. This study followed the Strengthening the Reporting of Observational Studies in Epidemiology (STROBE) reporting guidelines for cohort studies.

### Participants

All nontrainee ambulatory physicians in the practice network were eligible for inclusion in the analysis. The inclusion criteria from the previously reported feasibility study (>30 scheduled ambulatory clinical hours)^22^ were applied to each month’s data to best approximate which physicians worked exclusively in the ambulatory setting without teaching duties. These inclusion criteria also inherently exclude physicians decreasing their clinical effort in preparation to depart their position. To identify EHR use patterns associated with departure in time for future interventions, we excluded all data within 3 months prior of departure. Physicians with missing data for variables in the final model were excluded from the analysis.

### Data Sources

The primary data source for this study was EHR metadata from vendor-derived data platforms. EHR use data were collected from the Epic Signal platform, and scheduling data were collected from the Epic Clarity database. Signal tracks EHR user actions using a 5-second latency period, ie, once the user is idle for 5 seconds, tracking pauses until user actions resume. User actions are defined as any mouse action (ie, click, movement, scroll) or keyboard action (ie, keystrokes). This 5-second latency period was internally validated by Epic with a time-motion analysis (Provider Efficiency Technical Services team, email, April 6, 2020). A similar time-motion analysis has been performed on other Epic event logs.^12^ It is important to note that time on EHR tasks away from the computer will not reliably be captured, eg, a telephone call to patient to resolve an inbox message.

Physician demographic data, including termination status and departure date, were obtained from human resources rosters. These rosters did not include a specific reason for departure (ie, we are unable to determine whether a physician left their position to practice in another location, to leave the profession of medicine, or to retire).

### Measurements and Outcomes

*Physician characteristics* included age, gender, medical specialty, and termination date (if the physician departed their position during the study period). The primary outcome was physician turnover (ie, leaving one’s position in the practice network during the study period). To control for temporal trends in EHR use (eg, physician-level variability in EHR proficiency or efficiency over time), each month of data for each physician was assigned a monthly time trend variable corresponding to that physician’s study month number.

*Physician productivity measures* were analyzed to account for differences in clinical workload in terms of patient volume, intensity of work, and demand for a physician’s services. Patient volume was defined as the number of completed appointments per month. Intensity was defined as the number of completed appointments per scheduled hour of work. Demand was defined as the percentage of available patient time that was scheduled with appointments.

*EHR use measures* included the 5 core EHR use metrics that were feasible for implementation in vendor-derived EHR data platforms: total EHR time, normalized to 8 hours of scheduled patient time; work outside of scheduled clinical hours, normalized to 8 hours of scheduled patient time; encounter note documentation time, normalized to 8 hours of scheduled patient time; active time on inbox, normalized to 8 hours of scheduled patient time; and teamwork for orders, the percentage of a physician’s orders that are placed by other members of the care team.^22,24^ This final non–time-based metric was specifically proposed due to the extensive literature that team-based care can reduce professional burnout.^26,27,28,29,30,31,32^

### Statistical Analysis

Data were summarized using standard descriptive statistics for continuous variables and frequencies and percentages for categorical variables. The longitudinal data set consisted of monthly measures for each physician. Bivariate analyses were conducted using 2-sample *t* tests; associations for continuous variables and χ^2^, 2-sample proportions tests for categorical variables were used for cross-sectional data, such as physician characteristics or to compare the means of the mean values for all metrics at the physician level. Mean differences between groups based on repeated measures were also calculated by departure status using linear mixed models to adjust for within-physician association over time. To account for within-physician association due to the multiple observations for each physician, the associations between departure status, physician productivity, and EHR use patterns were examined on a monthly basis for each physician using generalized estimation equations (GEEs) for panel data (xtgee in Stata; family: binomial; link function: logit, exchangeable correlation structure) that allowed robust standard errors. Two models were built to model physician turnover as a function of EHR and productivity metrics: 1 using total EHR time as a variable of primary interest and another using time on inbox, note documentation, and work outside scheduled clinical hours, given that total EHR time is an approximate combination of these 3 metrics. Both models were adjusted by age, gender, physician specialty, and study month. Specialties were grouped into 3 categories: primary care, medical specialties, and surgical specialties. Since both patient volume and intensity include completed appointments per month, we chose to control for intensity in the final model (instead of volume alone) to control for additional dimensions of productivity, thereby potentially explaining more variance in physician turnover that is due to non-EHR factors. Variation in physician turnover that could be explained by the models (McKelvey and Zavoina *R*^2^) was computed using multivariable logistic regression models adjusted for correlated physician-level data (clustered by physician) since GEE did not provide a similar goodness-of-fit statistic. To obtain physician-level indicators of the estimating power of the models, area under the curve (AUC), positive and negative predictive values, sensitivity, and specificity were computed based on cross-sectional logistic regression models using means of the physicians across time for EHR metrics and productivity variables. The mean values of the total EHR time and time on inbox per month across physicians starting with the first month for each physician were illustrated using Lowess curves. All statistical analyses were conducted in Stata version 15 (StataCorp). Statistical significance was set at *P* < .05, and all tests were 2-tailed.

## Results

**Figure 1.**
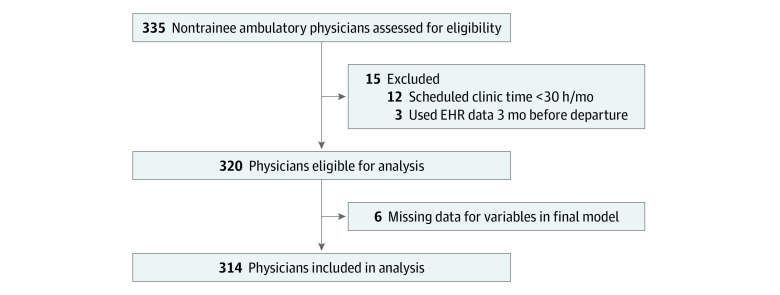
Study Flowchart EHR indicates electronic health record.

Among 335 physicians assessed for eligibility, 314 unique physicians (89.2%) met criteria and were included in the analysis, with a corresponding 5728 physician-months of data (Figure 1; Table 1); 123 (39%) were women, 100 (32%) were aged 45 to 54 years, and the turnover rate was 5.1%/year (10.2% total; 32 physicians departed their position during the study period). Six physicians were excluded due to missing data for the variables included in the multivariable models. The mean values for physician productivity were as follows: demand, 77% (95% CI, 75%-79%); patient volume, 206 completed appointments/month (95% CI, 197-215 appointments/month); and intensity, 2.6 patients/hour (95% CI, 2.5-2.6 patients/hour). Mean EHR use values were as follows: total EHR time, 5.5 hours (95% CI, 5.3-5.8 hours); note documentation, 1.9 hours (95% CI, 1.7-2.0 hours); work outside scheduled clinical hours, 0.8 hours (95% CI, 0.8-0.9 hours); time on inbox, 0.70 hours (95% CI, 0.66-0.74 hours); and teamwork, 23% (95% CI, 21%-25%).

**Table 1.** Physician Characteristics Stratified by Turnover Status

Characteristics	Physicians, No. (%)			*P* value^a^	Repeated measures, difference (95% CI)	*P* value^b^
	All (N = 314)	Departed (n = 32)	Stayed (n = 282)			
Gender						
Female	123 (39)	8 (25)	115 (41)	.08	NA	NA
Male	191 (61)	24 (75)	167 (59)			
Age, y						
<35	19 (6)	3 (9)	16 (6)	.06	NA	NA
35-44	65 (21)	9 (28)	56 (20)			
45-54	100 (32)	4 (13)	96 (34)			
55-64	79 (25)	7 (22)	72 (26)			
≥65	51 (16)	9 (28)	42 (15)			
Specialty						
Primary care	173 (55)	15 (47)	158 (56)	.58	NA	NA
Medical specialties	104 (33)	12 (38)	92 (33)			
Surgical specialties	37 (12)	5 (16)	32 (11)			
Productivity, mean (SD)						
Patient volume, completed appointments/mo	206 (82)	184 (97)	209 (80)	.11	−24.1 (−54.2 to 6.1)	.12
Demand, available appointments completed, %	77 (0.18)	66 (0.18)	78 (0.18)	<.001	−0.12 (−0.18 to −0.05)	<.001
Intensity, patients/h	2.6 (1.0)	2.6 (1.0)	2.6 (0.8)	.93	0.02 (−0.29 to 0.32)	.93
Months per physician	18.2 (6.0)	10.0 (5.9)	19.2 (5.2)	<.001	NA	NA
EHR use, mean (SD)						
Total EHR time, h^c^	5.5 (2.0)	5.1 (1.5)	5.6 (2.0)	.16	−0.5 (−1.2 to 0.2)	.16
Work outside scheduled clinical hours, h^c^	0.8 (0.8)	0.6 (0.5)	0.9 (0.8)	.12	−0.2 (−0.5 to 0.06)	.13
Note documentation, h^c^	1.9 (1.0)	1.7 (0.9)	1.9 (1.0)	.38	−0.2 (−0.5 to 0.2)	.41
Time on inbox, h^c^	0.7 (0.4)	0.5 (0.3)	0.7 (0.4)	.01	−0.2 (−0.3 to −0.04)	.01
Teamwork, %	23 (21)	17 (18)	21 (0.21)	11	−0.06 (−0.14 to 0.01)	.11

Abbreviations: EHR, electronic health record; NA, not applicable.

^a^ Based on mean of the mean values for each physician for values with multiple measures.

^b^ Differences for productivity and EHR use metrics were estimated using linear mixed models using repeated observations per physician.

^c^ EHR use measures were normalized to 8 hours.

**Figure 2.**
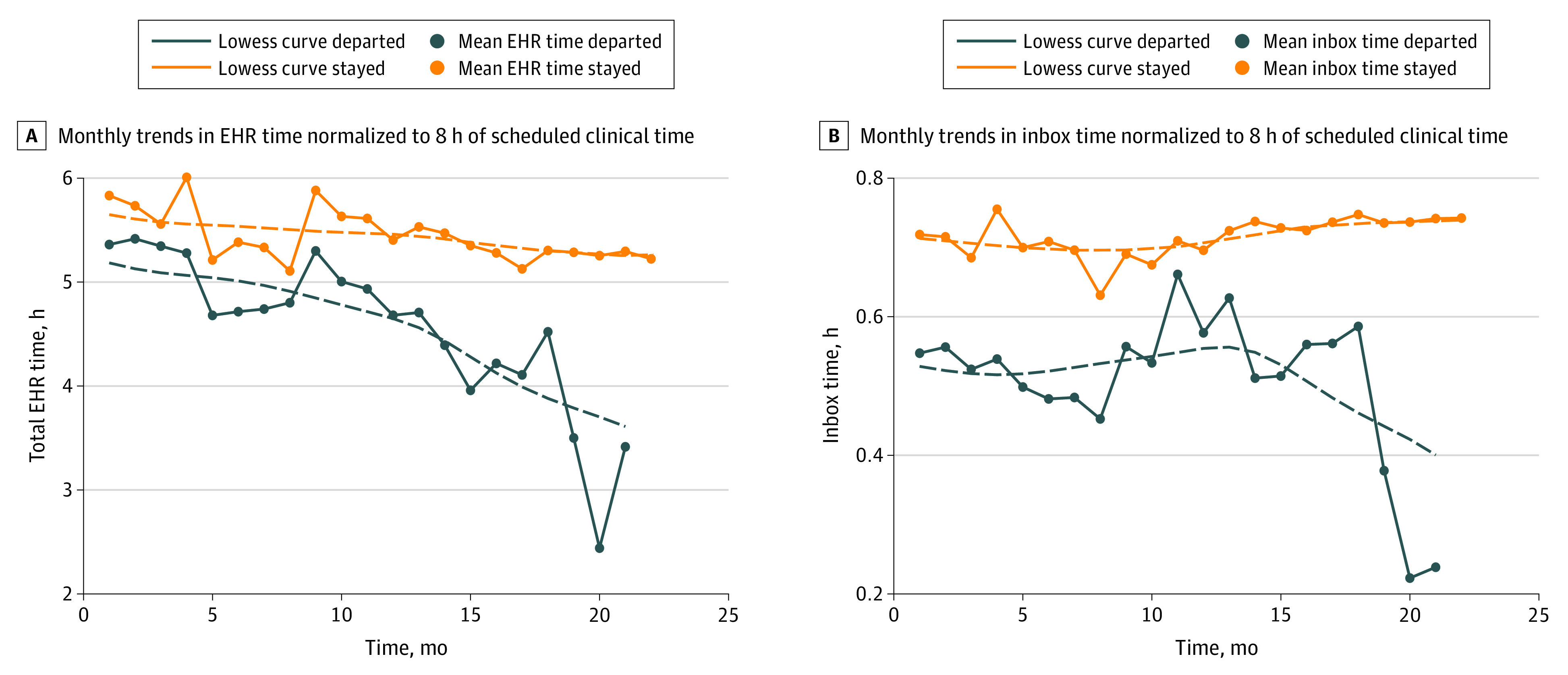
Monthly Trends in Core Metrics for Electronic Health Record (EHR) and Inbox Time, Normalized to 8 Hours of Scheduled Clinical Time and Stratified by Turnover Status During the Study Period

A wide range of ambulatory medical specialties were represented in the analysis, with a wide range of departure rates. The specialties with the highest turnover rates were other medical subspecialties (8 of 46 [17.4%]), surgical specialties (4 of 25 [16.0%]), gastroenterology (2 of 17 [11.8%]), and internal medicine (13 of 117 [11.1%]). In bivariate analyses, the following variables were associated with physician turnover: demand (difference between departed and remaining physicians, −0.12; 95% CI, −0.18 to −0.05; *P* < .001) and time on inbox (difference between departed and remaining physicians, −0.2; 95% CI, −0.3 to −0.04; *P* = .01) (Table 1). Monthly trends for total EHR time and time on inbox are presented in Figure 2, stratified by turnover status during the study period.

After controlling for physician age, gender, medical specialty, and study month in the generalized estimation equations model (Table 2), the following variables were associated with physician departure in the main model: time on inbox (odds ratio [OR], 0.78; 95% CI, 0.68-0.90; *P* = .001), teamwork (OR, 0.61; 95% CI, 0.46-0.82; *P* = .001), demand (OR, 0.56; 95% CI, 0.41-0.75; *P* < .001), and age 45 to 54 years vs 25 to 34 years (OR, 0.19; 95% CI, 0.04-0.95; *P* = .04). In the secondary model, total EHR time was also associated with physician turnover, with an adjusted OR of 0.96 (95% CI, 0.93-0.99; *P* = .01). McKelvey and Zavoina *R*^2^ was 0.35 for the main model. Adding EHR use metrics increased *R*^2^ from 0.21 to 0.35, indicating that the model accounts for 35% of variation in turnover and that EHR metrics accounted for 14% of variation in turnover. The estimating power was calculated as an AUC of 0.82, with a positive predictive value of 66.7%, negative predictive value of 90.9%, sensitivity of 12.5%, and specificity of 99.3%.

**Table 2.** Factors Associated With Physician Turnover in Generalized Estimation Equation Models for Longitudinal Data Among 5728 Months for 314 Nonteaching, Ambulatory Physicians

Variable	Model 1		Model 2	
	OR (95% CI)	*P* value	OR (95% CI)	*P* value
Gender				
Male vs female	1.95 (0.82-4.63)	.13	1.89 (0.80-4.48)	.15
Age, y				
25-34	1 [Reference]	NA	1 [Reference]	NA
35-44	0.82 (0.19-3.52)	.79	0.82 (0.19-3.50)	.78
45-54	0.19 (0.04-0.95)	.04	0.19 (0.04-0.94)	.04
55-64	0.41 (0.10-1.78)	.24	0.41 (0.10-1.76)	.23
≥65	0.86 (0.21-3.60)	.84	0.87 (0.21-3.63)	.84
Specialty				
Primary care	1 [Reference]	NA	1 [Reference]	NA
Medical specialties	1.31 (0.58-2.96)	.51	1.33 (0.59-2.98)	.49
Surgery or surgical specialties	1.62 (0.54-4.84)	.39	1.64 (0.54-4.94)	.38
Productivity				
Demand	0.56 (0.41-0.75)	<.001	0.56 (0.42-0.76)	<.001
Intensity	0.96 (0.82-1.14)	.66	0.97 (0.82-1.16)	.75
Study month	0.998 (0.997-0.999)	<.001	0.996 (0.995-0.997)	<.001
EHR use				
Time, normalized to 8 h				
Total EHR	NA	NA	0.96 (0.93-0.99)	.01
Time on inbox	0.78 (0.68-0.90)	.001	NA	NA
Note documentation	0.99 (0.94-1.05)	.83	NA	NA
Work outside scheduled clinical hours	0.96 (0.90-1.02)	.17	NA	NA
Teamwork	0.61 (0.46-0.82)	.001	0.60 (0.50-0.80)	.001

Abbreviations: EHR, electronic health record; NA, not applicable; OR, odds ratio.

## Discussion

### Key Results

Despite continued limitations to vendor-derived EHR use data platforms, the findings from this cohort study of physician turnover, productivity, and EHR use, as assessed by vendor-derived EHR use data platforms in a large ambulatory care network, indicate that physician productivity and EHR use metrics were associated with departure. Physician age, gender, and specialty were not associated with departure in bivariate or multivariate analyses. After controlling for these factors, less time on the EHR and inbox were both associated with physician departure. The direction of the association between these time-based EHR use metrics and physician departure was opposite of the hypothesized direction based on assumptions about the association between EHR usability and physician burnout. Lower rates of teamwork on order entry were associated with physician departure, indicating that teamwork may help to prevent physician turnover. This finding is consistent with evidence that team-based care can reduce professional burnout and optimize team performance.^26,27,28,29,30,31,32^ Taken together, these findings suggest that a refined, prospective model of vendor-derived EHR data could help to identify physicians at high risk of departure who might benefit from targeted team-based care interventions. Indeed, low demand for a physician’s service, time on the EHR and inbox, and rates of teamwork on orders may very well be lead indicators for physicians preparing to leave practice.

### Interpretation

Using vendor-derived EHR data to explore physician departure offers several advantages over traditional survey research. Vendor-derived EHR data are more objective, more available (given their automated collection without the burden of survey completion on the population being surveyed), and potentially more representative of the population being studied (since they are not subject to the response bias inherent in survey research). Even though we excluded the last 3 months of data for physicians who left their positions, the physician productivity metrics found to be associated with departure in this analysis are consistent with expectations that physicians leaving their position wind down their practice in anticipation of departure or may leave their position if there is not demand for their services. Physicians practicing in a productivity-based compensation network (like that studied here) who find themselves with consistently low patient volumes may choose to leave the network due to lower than expected income.

EHR use metric values for physicians who left their positions are more counterintuitive. If physicians who leave are in less demand or winding down their practice, they may have less EHR work to do or may be able to complete more of their EHR work during scheduled clinical hours. This could falsely decrease their EHR use time regardless of their efficiency or proficiency with EHR-related work. Alternatively, physicians who leave their practice could be more proficient with the EHR and, therefore, more marketable to move to a new position. This would suggest that more proficient EHR users are at an increased likelihood for departure. However, we are unable to conclude from this study any more details of the EHR proficiency of physicians who departed. Future studies could more definitively assess the association of burnout and departure with EHR use by linking survey data with EHR use and productivity metrics with more detailed information on departure. Furthermore, incorporating more detailed analysis of non–time-based EHR use patterns (eg, wrong-patient orders, number of clicks per patient) could help to better determine whether physicians who leave practice and spend less time on EHR work are indeed more proficient than their peers who stay in practice.^23^

Despite the effect size of inbox time and teamwork for orders, these metrics’ comparatively smaller *R*^2^ indicate that physician characteristics and productivity are likely more strongly associated with physician turnover than EHR use. This finding is consistent with a survey study of 1310 ambulatory clinicians, which found that work culture explained more variation in burnout than the EHR (*R*^2^, 17.6% vs 1.3%; *P* < .001).^33^ Similarly, physician-perceived EHR usability explained only 5.8% of burnout.^17^ Although 2 recent physician surveys reported that excessive EHR time was the most prevalent stressor associated with burnout (OR, 1.99; 95% CI, 1.21-3.27)^34^ and that physicians who were frustrated with their EHR had 2.4 times higher odds of burnout (95% CI, 1.6-3.7; *P* < .001),^35^ the findings from the present analysis suggest either a smaller contribution of the EHR to physician turnover or a more complex relationship between EHR use, burnout, and attrition. The counterintuitive direction of the association of time spent on EHR activities and physician departure warrants further investigation.

Additional future research could prospectively track physician productivity and EHR use patterns to identify physicians at risk of departure, thereby potentially allowing practice leaders to intervene and retain physicians at the highest risk of departure. Additional possibilities include strengthening model performance by: (1) extending study duration to allow earlier identification of physicians at high risk of departure in time for more robust retention attempts, (2) exploring additional practice dimensions that might be associated with departure, and (3) focusing model outcomes on reduction in professional effort or specific reasons for departure (ie, moving jobs, retiring, or leaving the profession). Specific practice dimensions worth exploring include (1) EHR dimensions related to note quality, teamwork on inbox, or inbox volume^19,20^; (2) panel size and complexity; (3) practice setting (including academic or telehealth); (4) medical specialty; and (5) temporal factors, such as the COVID-19 pandemic and vacation time. Additional work in this space would benefit from standardizing vendor-derived EHR data definitions in a way that is clinically relevant and important as well as qualitative exit interviews to provide further insight into existing data (eg, why physicians left their position) and the association between EHR use, burnout, and physician attrition.

### Limitations

This study has limitations. The main limitation was that the primary outcome—physician departure—did not include a reason, ie, whether a physician left their position to take a new job, retire, or leave the profession of medicine altogether. Subsequently, the study findings are more likely applicable to departure in general than a specific reason. This study was also subject to limitations related to vendor-derived data quality. For example, some data definitions were changed internally by the vendor during the study period. More importantly, vendor-derived data platforms could not reliably distinguish ambulatory care context and scheduled hours for teaching attendings. This limitation is not unique to this vendor or practice network.^22^ However, we cannot conclude whether the findings from this analysis are generalizable vs specific to the practice settings studied here based on local differences such as culture, climate, and compensation models.

## Conclusions

As the first study that we know of to use vendor-derived EHR data to model physician turnover, this study identified physician demand, inbox time, and teamwork on orders as key variables associated with physician departure. Counterintuitively, less time spent on the EHR (in particular on inbox management) was associated with physician departure and warrants further investigation. Standardizing vendor-derived data definitions (across and within vendor products) with better integration of clinical schedules^23,36,37^ could improve data validity and reliability. With greater data validity and reliability, future models could prospectively identify physicians at high risk of departure who would benefit from targeted interventions to improve retention.
